# Near UV-Vis and NMR Spectroscopic Methods for Rapid Screening of Antioxidant Molecules in Extra-Virgin Olive Oil

**DOI:** 10.3390/antiox9121245

**Published:** 2020-12-08

**Authors:** Giulia Vicario, Alessandra Francini, Mario Cifelli, Valentina Domenici, Luca Sebastiani

**Affiliations:** 1 BioLabs, Institute of Life Science, Scuola Superiore Sant’Anna, Piazza Martiri della Libertà 33, 56127 Pisa, Italy; g.vicario@santannapisa.it (G.V.); a.francini@santannapisa.it (A.F.); 2 Chemistry and Industrial Chemistry Department, University of Pisa, Via Moruzzi 13, 56124 Pisa, Italy; mario.cifelli@unipi.it

**Keywords:** extra-virgin olive oil, UV-Vis spectroscopy, ^1^H NMR, ^13^C NMR, pigments, secoiridoids, squalene, antioxidant properties, polyphenols

## Abstract

Several spectroscopic techniques have been optimized to check extra-virgin olive oil quality and authenticity, as well as to detect eventual adulterations. These methods are usually complementary and can give information about different olive oil chemical components with bioactive and antioxidant properties. In the present work, a well-characterized set of extra-virgin olive oil (cultivar *Frantoio*) samples from a specific area of Tuscany (Italy) were investigated by combining near UV-Vis absorption spectroscopy, ^1^H and ^13^C nuclear magnetic resonance (NMR) to identify and quantify different chemical components, such as pigments, secoiridoids and squalene, related to the nutritional and quality properties of olive oils. Moreover, the pigmentation index of olives, organoleptic and sensory properties, total phenolic compound contents and the lipidic fractions of olive oils were investigated. The results obtained are, finally, compared and discussed in order to correlate several properties of both olives and olive oils with specific features of the cultivation area.

## 1. Introduction

Spectroscopic methods are now widely considered efficient tools for food in terms of oxidative stability and quality but, also, authentication, due to their high sensitivity, rapidness and possibility to perform analyses directly on the sample without previous treatments [1,2,3]. Additionally, compared with standard chromatographic methodologies, spectroscopic techniques are often less time-consuming and less expensive. For these reasons, in the last decade, many spectroscopic techniques were optimized and used in combination with multivariate techniques to food analysis in terms of chemical composition, trace contaminant determinations, food quality and authenticity and fraud identifications [4,5]. Recent advances in the application of spectroscopic analytical methods for food quality assessment were reported in a recent review by Hassoun et al. [6] focusing on the application of nondestructive spectroscopic monitoring and process optimization during the production of meat and fish. Similarly, new highly technological spectroscopic approaches were reviewed by Wang et al. [7] concerning the trace analysis of persistent organic pollutants in different complex matrices, such as food ones. A very interesting review about the state-of-the-art of nuclear magnetic resonance methods applied to the quality assessment of a large variety of food and agricultural products was just published [8]. All these considerations are also valid for the case of olive oil. Several recent researches based on different spectroscopies have been carried out in order to determine the olive oil quality and authenticity [9,10]. A recent review about rapid and innovative instrumental approaches for oil quality assessment was published by Valli et al. [11].

Among the different categories of olive oils, different quality levels have been attributed, which also correspond to different economic values. This is probably one of the main reasons why extra-virgin olive oil (EVOO) is the most adulterated food matrix. Different frauds and adulterations, such as those based on the addition of different kinds of seed oils, refined oil or lower grade of olive oils, have stimulated the development of rapid, nondestructive and relatively cheap methods, such as several spectroscopic methods reported in several works [12,13,14].

Among the different spectroscopic techniques, infrared (IR) [15,16] and near infrared (near-IR) spectroscopies [17] have recently attracted much attention due to the very fast analyses and to the development of specific multivariate mathematical tools to rapidly detect typical olive oil adulterations [18]. These techniques are mostly centered on the identification and quantification of the fatty acid components and their derivatives [16,19].

Other spectroscopies applied to the study of olive oil are related to the absorption of UV-Vi light (UV-Vis absorption spectroscopy) and to fluorescence emission in the UV-Vis region (fluorescence spectroscopy). This last technique was only recently used to investigate the quality and authenticity of olive oil, mainly focusing on the chlorophyll contents in fresh oils versus not-fresh oils [20] and to the emission spectral profiles due to the polyphenols present in olive oil [21]. UV-Vis absorption spectroscopy is a technique able to detect with relatively high sensitivity single pigment contents in the visible range (from ~400 nm to ~800 nm) and other compounds present in olive oil, such as polyphenols, peroxides and other fatty acid derivatives due to the oxidation of olive oils, in the UV range (from ~230 nm to ~400 nm). UV-Vis spectroscopy combined with multivariate analyses was used to quantify the adulteration of extra-virgin olive oils (EVOOs) with low-grade oils [22]. It was also combined with electronic nose to verify the EVOOs’ geographical origin [23] and with other nonselective (near and mid-infrared spectroscopy) and selective techniques to characterize Protected Designation of Origin (PDO) olive oils [24]. This spectroscopic technique was also applied to thermal edible oil evaluation [25]. Recently, a novel method to identify and quantify the main pigment contents based on the deconvolution of near UV-Vis absorption spectra of olive oils was implemented [26,27,28] and applied to the study of EVOOs produced in different Mediterranean countries [10] and during different harvesting years [29]. The major advantages of near UV-Vis absorption spectroscopy for the quantification of pigments are the very fast analysis (few minutes), the very low expense of both instrument and analysis and the accuracy of pigment determinations comparable with conventional chromatographic techniques [10].

Finally, one of the most successful spectroscopies to characterize olive oils is nuclear magnetic resonance (NMR). In particular, ^1^H and ^13^C NMR techniques have been used to analyze both olive oil as it is (in the bulk) and olive oil extracts. These methods, in rapid development, are now considered valuable tools for olive oil analysis and characterization [9,30] Other magnetic nuclei, such as ^31^P, were used to investigate adulterations in olive oils by NMR [31,32]. ^1^H NMR spectra of olive oil analyzed without treatment or of its solvent extracts give information about all major and minor olive oil components [33,34,35]. A ^1^H NMR spectral analysis was indeed applied as the target analysis for the detection and quantification of phenolic compounds, diacylglycerols and for fatty acid composition determinations [9,34,36,37]. It was also employed in metabolic profiling and fingerprinting in order to detect adulterations [38], to assess geographical origins [35,39,40] or to define the employed harvest method [41]. ^13^C NMR spectroscopy was used mainly to provide valuable information about the acyl distribution and the acyl positional distribution of glycerol tri-esters in oils [40]. A combination of mono-dimensional and bi-dimensional NMR techniques were employed to follow the olive oil chain production [42], while multiple quantum NMR methods were optimized to explore the polyphenol contents in olive oil [43].

Considering the scarce literature about the combination of different analytical techniques to the in-depth characterization of the olive oil, quali-quantitative analyses using near-UV-Vis absorption and NMR spectroscopic methods can help to better understand the product composition and to bring innovation in the olive oil sector.

The aim of this study was to verify if spectroscopic techniques (often considered complementary to conventional methods) could discriminate the monovarietal EVOOs derived from a small geographical area (PDO “Terre di Siena”) for the antioxidant molecule composition.

## 2. Materials and Methods

### 2.1. Chemicals

Petroleum ether, deuterated chloroform, methanol, hexane, acetonitrile, Folin-Ciocalteu’s reagent, sodium carbonate, gallic acid, syringaldehyde (98% purity), 1-methoxy-2-(2-methoxyethoxy) ethane and deuterated chloroform (CDCl_3_) were purchased from Sigma-Aldrich (Milan, Italy). Water was purified by using a Milli-Q system (Merck-Millipore, Milan, Italy).

### 2.2. Sampling Area

The experimental orchard was in Southern Tuscany (Cetona, Italy). The farms participating in the ASIOL-BIOSi project (Applicazione di nuove Strategie e tecniche Innovative in OLivicoltura BIOlogica in provincia di Siena) provided olives and extra-virgin olive oil (EVOO) samples for sensory and chemical analyses. Three different locations were chosen: (1) 367-m altitude, 42.891° N 11.941° E, (2) 480-m altitude 42.854° N, 11.821° E and (3) 340-m altitude 42.918° N, 11.914° E. The three selected farmers produced a monovarietal EVOO (cultivar *Frantoio*), characterized by a high yield and high content of phenols.

During the maturation stage of olives (between the beginning of June and the end of December), the area was characterized by a maximum temperature of 41° C. Orchard management, including pruning, was conducted according to local commercial practice. Olive fruits (cultivar *Frantoio*) were sampled directly in the olive grown at the end of the ripening, just before the milling (13 December 2017) and were analyzed independently after 24 h from sampling. Fresh weight, pulp moisture, pulp fat content (Soxhlet extraction) and pigmentation index (Pi) [44] were determined. Olives were processed within 24 h from harvest in the same oil mill with a continuous cycle plant, which works without adding water, under controlled temperature with a maximum of 27 °C. EVOO samples were stored directly in dark glass bottles in a dark room at temperatures of 18–20 °C.

### 2.3. EVOO Analyses

Chemical and physical analyses for the classification of the produced oils established by the Commission Implementing Regulation (EU) No. 1348/2013 and by the PDO (Protected Denomination of Origin) specific regulation were done by the oil mill and external laboratories. All samples qualified for the PDO “Olio Terre di Siena” EVOO brand mark. Moreover, all samples respected the organic production regulation (European Council Regulation n. 834/2007).

Sensory analysis was carried out by a panel of 8 tasters according to the official standard (established by the International Olive Oil Council) on the 27 February 2018, and the results were organized in histograms.

Determination of total phenols content was done according Goldsmith et al. [45], with few modifications. Olive oils (5 g) were added to 15 mL of MeOH:H_2_O mixture (80/20, *v/v*). After vortexing the samples for 2 min, they were extracted in an ultrasonic bath for 15 min and centrifuged at 5600 rpm for 30 min at 4 °C (Allegra 64R, Beckma Coulter, Milan, Italy). About 2 mL of the supernatant phase was filtered through a 0.25-µm filter. An aliquot (300 µL) of this filtered phase was added to 300 µL of Folin-Ciocalteu’s reagent and left to react for 2 min. Na_2_CO_3_ 7.5% (2.4 mL) was added to the previous mixture and then left to react for 1 h in the dark at room temperature. A blank MeOH/H_2_O mixture (80/20, *v*/*v*) was prepared in the same way. Absorbance was measured at λ = 760 nm. A calibration curve was built using gallic acid as the standard (concentration between approximately 10 and 100 µg/mL). Results were expressed as gallic acid equivalents (GAE) g^−1^ fresh weight (FW). Samples were analyzed in triplicate.

Near-UV-visible (UV-Vis) absorption spectra collection does not require any sample preparation, as they were analyzed directly in the bulk. EVOO samples were put in a transparent quartz cuvette with an optical path of 0.5 cm. The spectra were recorded for each sample in triplicate between 220 and 800 nm by using a Jasco (Lecco, Italy) UV-Vis V-550 double-beam spectrophotometer; air was considered as the second beam background, and 1-nm resolution was used. The spectra acquired were normalized by subtracting the absorbance value at λ = 720 nm, where the sample does not absorb, to all the point in the spectra, before the spectral deconvolution for pigment quantification [26]. The main pigments (β-carotene, lutein and/or 9-cis-neoxantin, among carotenoids, and pheophytin A and B) and their content evolution in time (at 48, 168, 230 and 286 days after pressing) were studied. 

NMR experiments were performed using a Bruker (Milan, Italy) Avance DRX 400 spectrometer operating at a proton Larmor frequency of 401.36 MHz. ^1^H NMR spectral analyses were performed both on EVOO samples in the bulk and sample extracts to analyze the dominant lipidic component and the secoiridoids fraction, respectively. For a direct EVOO sample analysis, 50 µL of olive oil (without any internal standard) were added to 450 µL of deuterated chloroform and put in a NMR tube. ^1^H NMR spectra of EVOOs in the bulk were acquired with a 30° pulse on the ^1^H channel of 3.6 microseconds at 23 W, with a relaxation delay of 1.5 s and an acquisition time (FID) of 2 s in 32-k points. Number of scans was 256. Acquired spectra were processed using the TOPSPIN 3.5 program: they were zero-filled to 64 K, and the phase was corrected automatically, while the baseline was adjusted manually to optimize the signal integration. The peaks were selected manually, referring to the literature data [46,47], and their area was determined through deconvolution in the region of interest because signals often overlap, so it was difficult to do an accurate integration without deconvolution. Samples were studied in triplicate. Spectra of the replicas were normalized (ACD Labs NMR Spectroscopy softwar (Frankfurt, Germany), and the relative fatty acid composition was determined according to Guillen et al. [48]. 

Olive oil extraction was necessary to separate the hydrophilic phenolic compounds from the fat fraction of EVOOs. Sample preparation was done according to Karkoula et al. [49], with some modifications. About 4 g of the sample were added to 20 mL of hexane and shaken three times for 30 s, waiting 5 min between shakings. The result was a homogeneous phase, as the oil is soluble in hexane. The solution was mixed with 25 mL of acetonitrile, shaken three times for 30 s, waiting for 10 min between shakings, and then left until the phase is separated for approximately 1 h. Finally, the acetonitrile fraction was collected, 500 µL of internal standard (syringaldehyde, IUPAC name 4-hydroxy-3,5-dimethoxybenzaldehyde) were added and this mixture was evaporated under nitrogen flow. The residue of this extraction procedure was dissolved in 500 µL of deuterated chloroform (CDCl_3_) and transferred in an NMR tube. Samples were extracted in triplicate for standard deviation evaluation on the quantitative analysis. 

^1^H NMR spectra of the EVOO extracts were recorded by using a 30° pulse on the ^1^H channel of 3.9 μs at 29.92 W (90° pulse is 11.8 μs), with an acquisition time for the FID of 2 s (32-K points zero-filled to 64K before Fourier-transform) and a relaxation delay of 1.5 s. Number of scans was 400 for a total of about 16 min. The line broadening was set at 0.5 Hz for the FID Fourier-transform; the phase was adjusted automatically, while the baseline was corrected manually in the region of interest. Spectra were normalized. The peaks were selected manually, referring to the literature data [48,49]. The peaks area was determined through the deconvolution of the signals. Quantification of the identified compounds was done according the method proposed by Karkoula et al. [49,50].

^13^C NMR analyses were performed by using about 500 mg of olive oil dissolved in 500 µL of chloroform. Fifty microliters of internal standard (1-methoxy-2-(2-methoxyethoxy) ethane, named also *diglyme*) for squalene quantification were added. The protocol and the internal standard were chosen according Nam et al. [51]. ^13^C NMR spectra of EVOOs in the bulk were recorded by using a standard inverse-gated decoupling sequence: a 30° pulse on the ^13^C channel of 2.7 μs at 80.91 W (90° pulse was 8.22 μs) with an FID acquisition time of 0.6 s (64k points) and a relaxation delay d1 of 2.5 s in order to obtain a homogeneous magnetization recovery for all the signal in the spectrum. Proton decoupling (waltz16 90° pulse train of 90 ms at 0.51 W) was performed only during the ^13^C FID acquisition in order to remove the proton–carbon interactions. No proton irradiation during d1 delay was used in order to avoid the NOE (Nuclear Overhauser Effect) on carbon nuclei. Number of scans was 12,000 for a total acquisition time of about 10 h. Additionally, in this case, before the acquisition of all these spectra, lock, shim and tune were optimized. In this case, “auto-shim” was set. The spectra were zero-filled to 132 K, and line broadening was set at 1 Hz. Phase was adjusted automatically, while baseline was corrected manually. Peaks were selected manually, referring to the literature data [51,52]. Peak area was determined through the deconvolution of the signals. Squalene quantitative determination was done according Nam et al. [51]. 

### 2.4. Statistical Analysis

Data were expressed as means ± standard deviation (SD) for the triplicate runs. Moreover, the coefficient of variation (CV%) was determined in order to assess the precision and the repeatability of the method (quantitative NMR (qNMR)). The one-way ANOVA was then used to examine differences between the mean values. Bonferroni’s multiple comparison test was used to establish the significance of the obtained differences (*p* < 0.05). Statistical analysis was performed in PRISM, GraphPad 5 (San Diego, CA, USA).

## 3. Results

### 3.1. Olive Fruits Analysis

**Figure 1:**
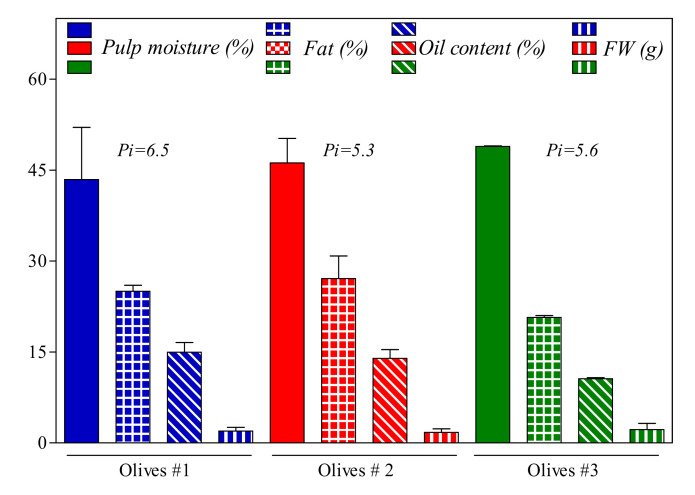
Change of some fruit physical and chemical characteristics of *Frantoio* olives harvested in three different location areas of Cetona (Tuscany, Italy). Pi: Pigmentation index; FW: fresh weight.

Olive fruits used for the oil production presented fresh weight and pulp moisture (Figure 1) between 1.7 and 2.2 g and 42% and 49%, respectively. Pulp fat content at the time of harvest was in the range of 20–27% of the total olive weight. The pigmentation index was lower in the olive group 2 compared to olive groups 1 and 3, but none of these differences was significant. 

### 3.2. EVOO Analyses

**Figure 2:**
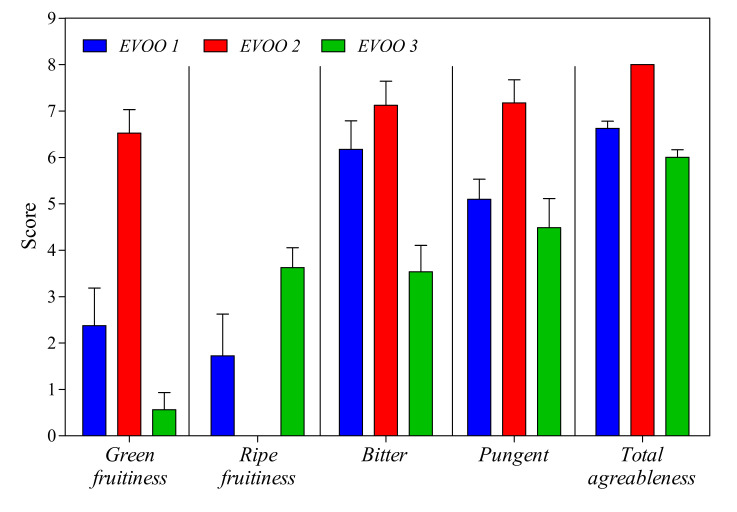
Bar plot of quantitative descriptive attributes of the three extra-virgin olive oils (EVOOs) studied.

All the oils were obtained in the same oil mill, and the mandatory chemical analyses for “extra-virgin” category assignment were performed in the mill. Organoleptic analysis results were reported in Figure 2. No taste–olfactory defects were detected, confirming the “extra-virgin” category, but oils differ in terms of green fruitiness, bitter and pungent sensations and persistence of the aroma and taste. According to the descriptions, EVOO 2 is surely the most interesting: pungent and bitter notes are the most relevant perceptions, but also, a green fruitiness was detected. EVOO 1 and EVOO 3 show flatter profiles due to the reduced pungent/bitter gustative perceptions and the minor intensity of the fruitiness olfactory notes (green fruitiness was practically not relevant). 

As phenols are related to pungent and bitter perceptions and have important health properties, the total amount of phenols was determined by a Folin-Ciocalteu assay. The total phenols content results (Table 1) were significantly different in the EVOOs; EVOO 2 showed the highest content of total phenols compared to EVOO 1 and EVOO 3 and, in particular, +35% and +44%, respectively.

**Figure 3:**
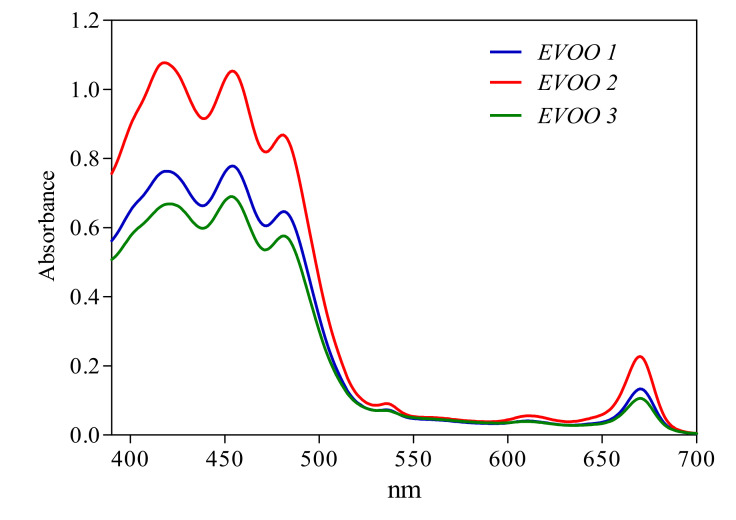
Near-UV-Vis absorption spectra of the three EVOO samples (EVOO 1: blue, EVOO 2: red and EVOO 3: green) in the range of wavelengths from 390 to 700 nm.

EVOO pigment contents are generally related to nutritional and stability properties. The acquisition of the near UV-Vis absorption spectra of EVOO samples is extremely fast (1 to 2 min), and the quantitative analysis of the experimental spectra were performed by using a mathematical approach [26,27]. As expected, the near UV-Vis absorption spectral profiles (Figure 3) were inherent to extra-virgin olive oils and were similar for all the samples. However, considering the area below the curve and the absorbance values, it is evident also from a qualitative comparison that EVOO 2 contained more pigments with respect to EVOO 1 and EVOO 3. Among the various mathematical models tested, the best model for a quantitative description of EVOO pigments was the five-pigments (six-functions) model specific for olive oils after some months of storage (the R-square values obtained by using four different models are reported in the Supplementary Materials). This model allows the quantification of β-carotene, pheophytin-A, pheophytin-B, lutein and cis-neoxanthin, and it is very accurate (see Tables S1–S3 in the Supplementary Materials).

**Figure 4:**
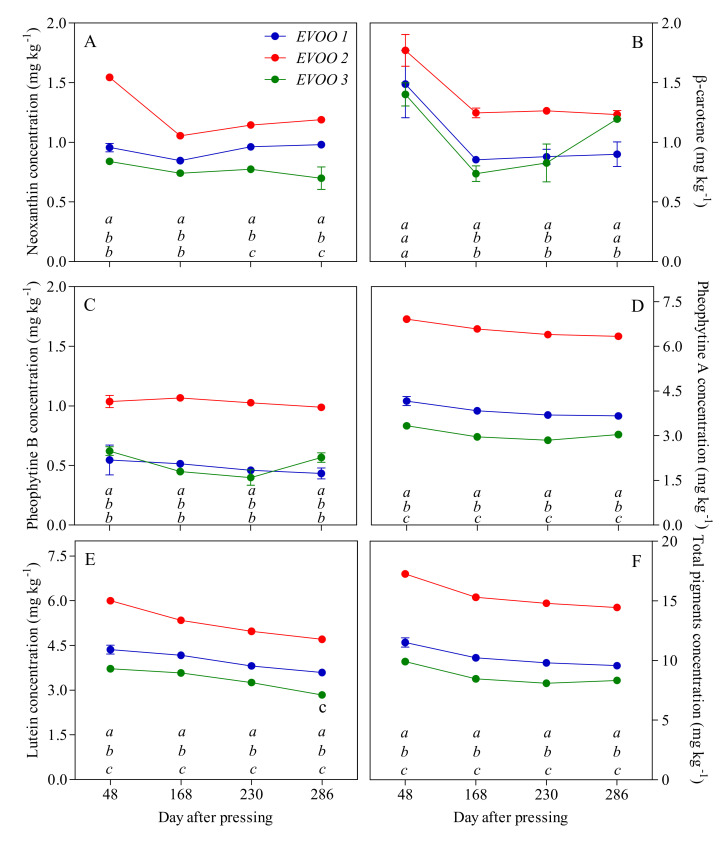
Pigment concentrations (mg kg^−1^) in EVOOs at different times of storage (days after pressing). (**A**) Neoxanthin; (**B**) β-carotene; (**C**) Pheophytin B; (**D**) Pheophytin A; (**E**) Lutein; (**F**) Total pigments concentrations. For each sampling data, different letters indicate significant differences among EVOOs. One-way ANOVA (Bonferroni post-test correction, *p* < 0.0001).

The time evolution of pigment contents during the storage of the oil is reported in Figure 4. After 48 days of storage (first sampling), the total pigment contents were significantly different in the three EVOOs of interest: EVOO 2 has higher amounts with respect to EVOO 1 and EVOO 3. With respect to the initial pheophytins fraction, EVOO 2 shows approximately double the amount of pheophytin A and pheophytin B with to respect EVOO 1 and EVOO 3, although the pheophytin A content was significantly different among the three samples. Considering carotenoids, no significant differences were detected in the β-carotene initial content, while cis-neoxanthin was more abundant in EVOO 2. Major differences can be observed in the lutein content. After 168 days of storage, total pigment contents were decreased in all the samples (−11% for EVOO 1 and EVOO 2 and −15% for EVOO 3). At the end of storage (286 days), losses in the pigments reached −17% in EVOO 1 and −16% in EVOO 2 and EVOO 3. The degradation of carotenoids and pheophytins occurred differently. Considering pheophytin A and pheophytin B (Figure 4C,D), in EVOO 1, we noted a reduction of −12% and −21%, respectively, while, in EVOO 2 and EVOO 3, the reduction was considerably lower (−8% and −5%, respectively, in EVOO2 and −9% in EVOO 3). With respect to the carotenoids, the amount of lutein was gradually reduced during the storage, reaching −17%, −21% and −23% in EVOO 1, EVOO 2 and EVOO 3, respectively, at the end of the storage (Figure 4E). As concern β-carotene and cis-neoxanthin, the decrease did not have a linear trend differently from lutein, which represents the main carotenoid in olive oil. After 286 days, the β-carotene content was reduced by −40%, −30% and −15% in EVOO 1, EVOO 2 and EVOO 3, respectively, but its amount in EVOO 3 behaved differently during the storage (Figure 4B). The cis-neoxanthin content, which is the minor carotenoid, was basically constant in EVOO 1 but consistently lower in EVOO 2 and EVOO 3 (−23% and −17%, respectively) (Figure 4A).

In the middle of the storage period, the ^1^H NMR spectra of EVOO samples in the bulk were acquired directly, dissolving the EVOO in deuterated chloroform. After normalization, the replicas of each sample showed the same spectral shape and the same proportions between signals, confirming the high repeatability of this ^1^H NMR technique. The three EVOO spectra were overlapped in order to observe qualitative differences between the EVOOs of interest. Overall, qualitatively, it is possible to observe that the three EVOO samples are very similar in saponifiable fraction signals. Linolenic, linoleic, oleic and saturated acids were quantified according the formulas proposed by Guillen et al. [48] (see Table S4 in the Supplementary Materials). No significant differences were detected. Additionally, diacylglycerols signals appeared qualitatively similar in the three samples (see Figure S1 in the Supplementary Materials).

**Figure 5:**
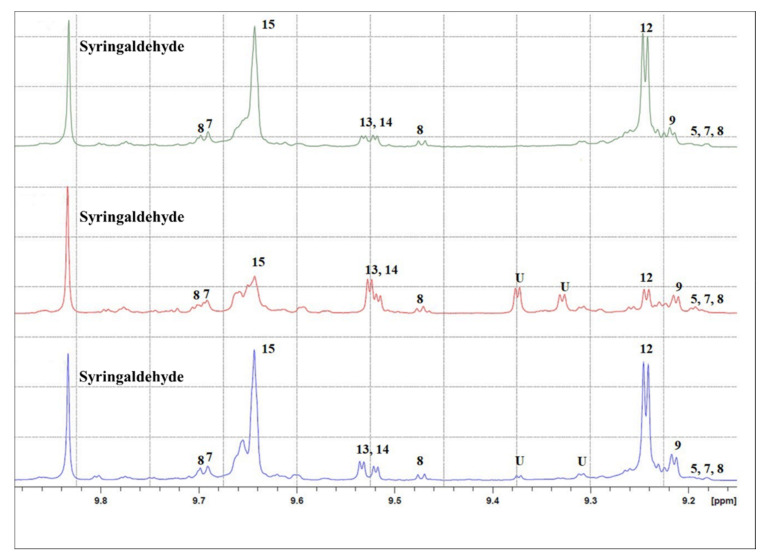
^1^H NMR spectra of EVOO 1 (blue), 2 (red) and 3 (green) in the 9.15–9.9 region. Syringaldehyde is the internal standard for quantification. Signals were identified according the literature data, and numeration refers to: (5) oleomissional, (7) 5S, 4R-oleuropeindial, (8) 5S, 4S-oleuropeindial, (9) oleacein (3,4-DHPEA-EDA), (12) oleocanthal (p-HPEA-EDA), (13) p-HPEA-EA (ligstroside aglycone), (14) 3,4-DHPEA-EA (oleuropein aglycone), (15) elenolic acid and U = unknown signal. More details in Table S5.

^1^H NMR spectra of the EVOO extracts were acquired to determine the secoiridoid chemical composition. The ^1^H NMR spectra of EVOO 1 and EVOO 3 appeared more similar than that of EVOO 2, especially concerning oleacein (identified by number 9 in Figure 5) and oleocanthal signals (identified by number 12 in Figure 5). EVOO 2 shows more intense oleuropein and ligstroside aglycones signals (identified in Figure 5 by the numbers 13 and 14, respectively) and a variety of not-yet-characterized signals between 9.3 and 9.4 ppm.

Moreover, the squalene content was determined through the analysis of ^13^C NMR spectra acquired in the bulk. Table 2 shows the results of the quantification of secoiridoids (oleacein, oleocanthal, oleuropein and ligstroside aglycones) and squalene.

As expected, the squalene content was higher compared to other minor compounds and the quantification was quite accurate (maximum coefficient of variation (CV) = 17.8%). No significant differences in squalene content were detected between the oils. Considering secoiridoids, the oleacein content was comparable between the oils, even if its quantification was the most variable considering the CV (24%). Oleocanthal was more abundant in EVOO 1 and EVOO 3 compared with EVOO 2. The ligstroside aglycone content was comparable between oils, while oleuropein aglycone was higher in EVOO 2 followed by EVOO 1 and then EVOO 3. Oleocanthal and oleuropein/ligstroside aglycones determination was more accurate (CV% max = 12.7%). The total aglycones content was also considered, because there is no agreement in the assignment of ligstroside and oleuropein aglycones signals. EVOO 2 showed the highest content in terms of total aglycones content.

### 3.3. PCA Results

**Figure 6:**
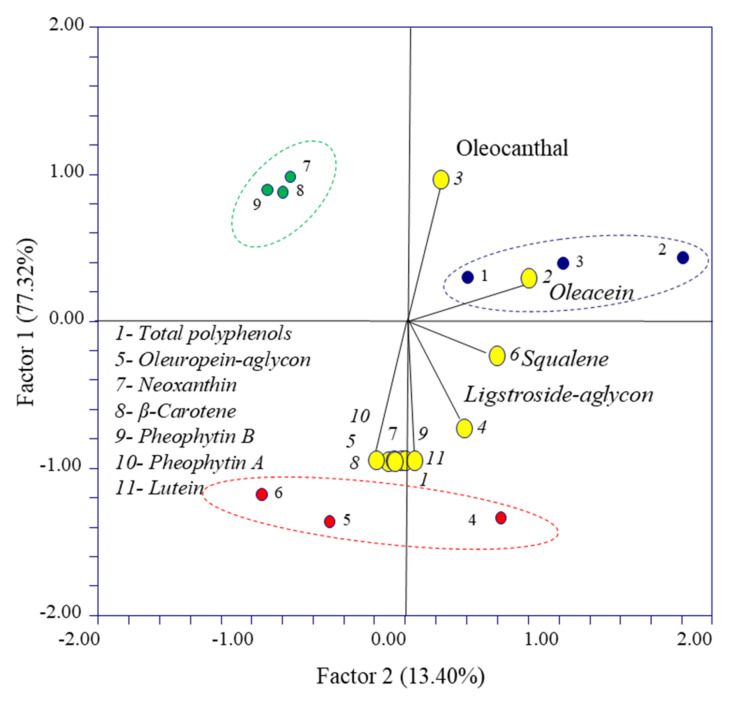
Bi-plot of the principal component analysis computed with the main results of the experiment. Data obtained from different EVOOs are reported with different colors: EVOO 1 blue, EVOO 2 red and EVOO 3 green.

A principal component analysis was performed to explore the data distribution patterns (total polyphenols, secoiridoids, squalene and pigments) and visualize the potential relationship among the studied variables and EVOOs. Figure 6 shows the bi-plot graph for the scores and loadings obtained. EVOO 1 and EVOO 3 obtained from olives with Pi = 6.5 and Pi = 5.6, respectively, are distributed on the top side of the graph, while the EVOO 2 obtained from olives with Pi = 5.3 is distributed on the bottom side of the graph. In order to detect important variables, a two-dimensional scatterplot of X-loadings for the first two components from the PCA is represented also in Figure 6. Similarly, Table 3 shows the loading factors of the PC1 and PC2 variables. The values with the greatest factor loading for PC1 are total polyphenols, oleocanthal, oleuropein-aglycon, neoxanthin, ligstroside–aglycon, β-carotene, pheophytin-B, pheophytin-A and lutein. Likewise, the variables showing a greater factor loading of the PC2 component were oleacein and squalene. The PCA analyses indicated that the compound oleacein is correlated with EVOO 1, while the attributes of total polyphenols, oleuropein–aglycon, neoxanthin, β-carotene, pheophytin B, pheophytin A and lutein are correlated with the EVOO 2. Finally, the data corresponding to the EVOO 3 sample did not cluster with any variable.

## 4. Discussion

EVOOs are food products that present a high variability of physical/chemical properties and their chemical and physical characterizations are usually performed by using different analytical methods. Several new and innovative techniques were recently optimized following the increased demand to replace traditional analytical methods with instrumental and, in particular, spectroscopic ones. The advantages to use spectroscopic methods are related to the speed of analysis, the possibility to analyze food matrices rich on antioxidant compounds without chemical or physical treatment and, in many cases, the high sensitivity combined with low cost. Chemical and food industries are looking forward to innovative instrumental techniques, such as spectroscopic methods for the qualitative and quantitative analyses of EVOOs. For instance, in the last years, the interest of a rapid and accurate quantification of pigments in the olive oil matrix has grown, and various spectrophotometric methods have been proposed [26,27]. Although analytical techniques aim to characterize the final product, the knowledge of the quality of olives at harvesting time, such as the area of cultivation and ripening stage, can help to make more accurate qualitative and quantitative analyses of EVOOs.

Considering the growing conditions, Mansour et al. [53] observed differences in the olive oil antioxidant chemical compositions of two local cultivars (*Chemlali* and *Neb Jmel*) grown in different areas: they detected higher olive oil total phenol contents at a higher altitude of cultivation. Similarly, Aguilera et al. [54] characterized EVOOs from the *Frantoio* cultivar grown in two different locations of Andalusia, showing that phenol contents in olive oils were higher at a higher altitude. In the present study, similar results were obtained from EVOO 2 that have been produced from olive groves located at 480 m above the sea level, while EVOO 1 and EVOO 3 derive from olive groves located at 367 m and 340 m, respectively. This finding brings us to suppose that the different altitudes can influence the phenol EVOO composition. 

Moreover, the slightly lower pigmentation index of olives of EVOO 2 detected at harvest time is in-line with the higher concentrations of total polyphenols detected in olive oil. As reported by Bengana et al. [55], there is an influence of olive ripeness on the chemical properties and phenolic composition of *Chemlali* extra-virgin olive oil. Chlorophylls and polyphenols tend to decrease in olive oil from advanced maturation stages of olives. 

Some authors reported that olive ripeness has a strong impact on olive oil descriptors [56,57]. The small differences in pigmentation indices found among the three EVOOs in the present study are in agreement with the organoleptic properties. Data about green fruitiness highlights the higher score found in EVOO 2 with respect to the others. The literature reports that this attribute decreases with the ripening stage, and it tends to entirely disappear in the oil from fruits with high Pi [56]. Considering the pigments contribution to olive oil visual characteristics, many adulterations try to confuse consumers, mimicking the olive oil color by adding colorants [58]. Although is not our case, the near-UV-Vis method applied in this work has high potentialities for detecting adulterations of olive oil due to pigment additions. Moreover, chlorophylls and carotenoids are antioxidant compounds, and their quantification in olive oils stored in dark conditions is important for the quality and authenticity of EVOO samples. Furthermore, the near-UV-Vis spectroscopic method for EVOO analyses can also be useful to discriminate different olive oils. In this context, a recently published method [26,28] showed that the near-UV-Vis absorption spectrum of EVOO can be reproduced by the linear combination of four/five pure, mainly pigment spectral contributions. This method, applied to the present case, shows a general lower pigment content with respect to the data reported in the literature for EVOOs from the cultivar *Frantoio* [28], and this is probably due to the late harvest time of olives. Moreover, and more interesting, the total pigment contents were significantly different in the three EVOOs studied, EVOO 2 being the one with the highest, as also indicated by PCA analyses and in agreement with sensory properties of the EVOOs analyzed in this work.

Secoiridoids, like oleuropein and its derivates, belonging to the antioxidant class of polyphenols, have potential applications for several cancer treatments [59]. It is well-known that their amounts in EVOOs depend on many factors, such as the cultivar, ripening degree, pedoclimatic condition and irrigation. The ripening degree influences the oleuropein content; in fact, oleuropein increases during the first ripening stages (green olives) and, later, decreases significantly due to the polyphenol oxidase activity [60,61]. During ripening, the oleuropein decreases, because it is recycled to dimethyl oleuropein and elenolic acid glucoside [62]. Other interesting secoiridoid derivatives are aglycon forms of the secoiridoid glucosides produced during oil extraction [63]. A specific secoiridoid compound—namely, oleocanthal—firstly described by Montedoro et al. [64], is a promising antioxidant agent in the prevention of chronic diseases related to persistent conditions of inflammation and oxidative stress, attracting an increasing interest on its quantification [65,66]. Using ^1^H NMR, the secoiridoid contents in the three EVOO samples were determined, showing that EVOO 2 has the highest amount of aglycon isomers of oleuropein (see Table 2), bioactive compounds associated with the bitter and pungent taste of olive oil [67,68]. This result agrees with the higher score of EVOO 2 of this parameter compared to EVOO 1 and EVOO 3, as reported in Figure 2.

Concerning oleocanthal, together with oleacin, its determination again discriminates EVOO 2 from the other two samples, EVOO 1 and EVOO 3, as reported in Table 3.

With the help of NMR spectroscopy, the olive oil matrix could also be directly investigated in order to assess the fatty acid profile, triacylglycerol composition, presence of diglycerides and trans-fatty acids, waxes and sterols profile [33,69,70]. In this context, we applied the method proposed by Nam et al. [51] for the direct quantification of squalene through ^13^C NMR spectroscopy to our three EVOO samples. As shown in Table 2, no significant differences were revealed among the three EVOOs, but the amount of squalene detected in the present work are comparable to those found by Nam et al. [51] in the EVOO samples.

## 5. Conclusions

The presence of antioxidant molecules and their abundance in EVOOs strongly contributed to the organoleptic properties of the final product. The spectroscopic methods (UV-Vis and NMR) used in this work allowed to identify and quantify ten different olive oil chemical components with bioactive and antioxidant properties. In particular, five main pigments (neoxanthin, β-carotene, pheophytin-B, pheophytin-A and lutein) were determined and studied from the olive oil production to a long period of storage by using a fast and very cheap UV-Vis spectroscopic method. Four different secoiridoids (oleacein, oleocanthal, ligstroside–aglycon and oleuropein–aglycon) and squalene were quantified by using different NMR methods based on the ^1^H and ^13^C nuclei. Using compound types and abundance information, a discrimination analysis of monovarietal EVOOs derived from a small geographic area was achieved, proving the validity of spectroscopic methods for EVOO antioxidant identification.

## Figures and Tables

**Table 1:** Total phenol contents in three different extra-virgin olive oils (EVOOs) (mg kg^−1^ fresh weight (FW)). Different letters represent differences following one-way ANOVA, Bonferroni post-test *p* < 0.05. Data represent means ± standard deviation of three replicate.

	Total Phenols Content
**EVOO 1**	129.64 ± 3.22 ^b^
**EVOO 2**	200.88 ± 1.90 ^a^
**EVOO 3**	111.03 ± 2.38 ^c^

**Table 2:** Oleacein, oleocanthal, oleuropein and ligstroside aglycones and squalene concentration (mg kg^−1^) in EVOO extracts, as obtained from ^1^H NMR spectral analyses. Data are shown as mean ± SD. One-way ANOVA was performed (Bonferroni test correction *p* < 0.05). For the same compound, different letters indicate differences among the EVOOs.

	EVOO 1	EVOO 2	EVOO 3
Ligstroside aglycone	27.9 ± 1.43	29.9 ± 2.27	25.4 ± 1.23
Oleuropein aglycone	39.6 ± 2.05 ^b^	67.8 ± 1.80 ^a^	26.2 ± 2.52 ^c^
Total aglycones content	67.5 ± 2.25 ^b^	97.7 ± 2.56 ^a^	51.5 ± 1.56 ^c^
Oleacein	43.1 ± 10.51	31.0 ± 4.37	31.9 ± 1.64
Oleocanthal	207.4 ± 13.97 ^a^	40.0 ± 2.34 ^b^	193.6 ± 24.56 ^a^
Squalene content	3161 ± 554	2906 ± 248	2488 ± 78

**Table 3:** X-loadings for the variables with respect to the principal components 1 and 2.

Variables		PC1	PC2
1	*Total polyphenols*	−0.998	−0.051
2	*Oleacein*	0.261	0.861
3	*Oleocanthal*	0.937	0.289
4	*Ligstroside* *–* *aglycon*	−0.769	0.439
5	*Oleuropein* *–* *aglycon*	−0.990	0.037
6	*Squalene*	−0.270	0.650
7	*Neoxanthin*	−0.991	0.060
8	*β-Carotene*	−0.988	−0.012
9	*Pheophytin-B*	−0.987	−0.129
10	*Pheophytin-A*	−0.997	−0.007
11	*Lutein*	−0.991	0.084

## Supplementary Materials

The following are available online at https://www.mdpi.com/2076-3921/9/12/1245/s1: Table S1, Table S2 and Table S3: Report the R-square obtained by fitting with four models the near-UV-Vis spectra of EVOO1, EVOO2 and EVOO3, respectively. Table S4: Reports the values of fatty acid components in the three EVOO samples. Table S5: Shows the assignment of the ^1^H chemical shift of several compounds investigated in this work. Figure S1: Shows a selection of ^1^H NMR spectra recorded in bulk (diacylglycerols signals) on the three EVOO samples.
