# Supplementary material for: Near UV-Vis and NMR Spectroscopic Methods for Rapid Screening of Antioxidant Molecules in Extra-Virgin Olive Oil
Source: Antioxidants (Basel). 2020 Dec 8;9(12):1245. doi: 10.3390/antiox9121245 (PMC7764626; doi:10.3390/antiox9121245)
Supplement: Supplementary file 1 [file antioxidants-09-01245-s001.pdf]

SUPPLEMENTARY MATERIALS

Results of near UV-Vis spectroscopy: comparison between different fitting models

The original near-UV spectra of olive oils can be denconvoluted by using different models (Domenici et al 2014, Buti, 2016, Borrello et al., 2019). Models 1 and 2 are more appropriate for not fresh olive oils, while models 3 and 4 are more appropriate for fresh olive oils.

- Model 1 includes four main pigments (β-carotene, lutein, pheophytin a and pheophytin b);
- Model 2 includes five main pigments (β-carotene, lutein, cis-neoxanthin, pheophytin a and pheophytin b).
- Model 3 includes four main pigments (β-carotene, lutein, chlorophyll a and chlorophyll b);
- Model 4 includes five main pigments (β-carotene, lutein, chlorophyll a and chlorophyll b).

Table S1. Average R-square values of EVOO 1 in the four models at different sampling time. Model 1 corresponds to four pigments' model (EVOO after some months of storage); Model 2 corresponds to five pigments' model (EVOO after some months of storage); Model 3 corresponds to four pigments' model (EVOO fresh pressed); Model 4 corresponds to five pigments' model (EVOO fresh pressed).

Days after pressing	48	168	197	230	286
			Average R-square		
**Model 1**	0.996405	0.997816	0.997555	0.997570	0.997449
**Model 2**	0.996866	0.998881	0.998539	0.998975	0.998859
**Model 3**	0.995768	0.997144	0.996685	0.996816	0.996649
**Model 4**	0.996663	0.998668	0.998244	0.998758	0.998640

Table S2. Average R-square values of EVOO 2 in the four models at different sampling time. Model 1 corresponds to four pigments' model (EVOO after some months of storage); Model 2 corresponds to five pigments' model (EVOO after some months of storage); Model 3 corresponds to four pigments' model (EVOO fresh pressed); Model 4 corresponds to five pigments' model (EVOO fresh pressed).

Days after pressing	48	168	197	230	286
			Average R-square		
**Model 1**	0.996768	0.997095	0.997096	0.997101	0.997069
**Model 2**	0.998016	0.998097	0.998172	0.998260	0.998291
**Model 3**	0.996066	0.996120	0.995987	0.996034	0.995939
**Model 4**	0.997927	0.997695	0.997731	0.997852	0.997866

Table S3. Average R-square values of EVOO 3 in the four models at different sampling time. Model 1 corresponds to four pigments' model (EVOO after some months of storage); Model 2 corresponds to five pigments' model (EVOO after some months of storage); Model 3 corresponds to four pigments' model (EVOO fresh pressed); Model 4 corresponds to five pigments' model (EVOO fresh pressed).

Days after pressing	48	168	197	230	286
			Average R-square		
**Model 1**	0.995166	0.997827	0.997460	0.997673	0.996291
**Model 2**	0.995335	0.998771	0.998536	0.998721	0.996535
**Model 3**	0.994337	0.997126	0.996596	0.996942	0.995237
**Model 4**	0.995033	0.998543	0.998262	0.998493	0.996057

Results of ^{1}H NMR of olive oils in the bulk:

From the analysis of ^{1}H NMR spectra of olive oils in the bulk (see a selection in Figure S1) the following percentages of fatty acids are obtained for the EVOO samples.

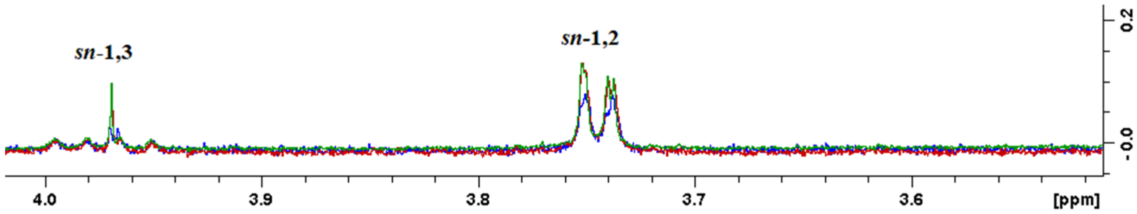

Figure S1. ^{1}H NMR spectral region showing diglycerides signals (DGs). Blue line corresponds to EVOO 1, red line to EVOO 2 and green line to EVOO 3. 1,2- diglyceride groups (*sn*-1,2) and 1,3-diglyceride groups (*sn*-1,3).

Table S4.1 Fatty acid data are shown as mean ± standard deviation. *Saturated acids refer mainly to palmitic and stearic acids.

Fatty acids	EVOO 1	EVOO 2	EVOO 3
**Linolenic acid (%)**	0.52±0.03	0.51±0.02	0.49±0.03
**Linoleic acid (%)**	7.56±0.08	7.99±0.12	7.91±0.10
**Oleic acid (%)**	78.11±0.57	78.80±1.37	79.59±1.24
**Saturated* acids (%)**	13.80±0.59	12.69±1.43	12.08±1.36

Table S5. Chemical shifts (in ppm) assignment of the ^1H NMR signals in $CDCl_3$ of protons of some phenolic compounds from literature data (modified from Ruiz-Aracama et al., 2017; Karkoula et al., 2012).

Signal	Compound	Chemical shift (ppm)	Multiplicity	Funcional Group
**5**	Oleomissional	7.360	dd	=C**H**-OH (C-3)
		9.190-9.205	os	-CHO (C-1)
		11.780	d	=CH-O**H** (C-3)
**6**	Oleokoronal	7.386	dd	=C**H**-OH (C-3)
		9.207-9.222	os	-CHO (C-1)
		11.764	d	=CH-O**H** (C-3)
**7**	5S, 4R- oleuropeindial	9.190-9.205	os	-CHO (C-1)
		9.670	d	-CHO (C-3)
**8**	5S, 4S- oleuropeindial	9.190-9.205	os	-CHO (C-1)
		9.448	d	-CHO (C-3)
**9**	Oleacein (3,4-DHPEA-EDA)	9.209	d	-CHO (C-1)
		9.22	d	-CHO (C-1)
		9.615-9.645	os	-CHO (C-3)
**10**	5S, 4R- ligstrodial	9.207-9.222	os	-CHO (C-1)
		9.680	d	-CHO (C-3)
**11**	5S, 4S- ligstrodial	9.207-9.222	os	-CHO (C-1)
		9.452	d	-CHO (C-3)
**12**	Oleocanthal (p-HPEA-EDA)	9.223	d	-CHO (C-1)
		9.23	d	-CHO (C-1)
		9.615-9.645	os	-CHO (C-3)
**13**	p-HPEA-EA (ligstroside	9.499	d	-CHO (C-1)
	aglycone)	9.52*	d	-CHO (C-1)
**14**	3,4-DHPEA-EA (oleuropein	9.504	d	-CHO (C-1)
	aglycone)	9.50*	d	-CHO (C-1)
**15**	Elenolic acid	9.615-9.645	os	-CHO (C-1)
	Unknown compounds			
**16**	Unknown	9.310	d	-CHO
**17**	Unknown	9.355 (9.37)	d	-CHO

Abbreviations: 3,4-DHPEA-EDA: dialdehidyc form of decarboxymethyl elenolic acid linked to hydroxytyrosol (3,4-dihydroxyphenylethanol), p-HPEA-EDA: dialdehidyc form of decarboxymethyl elenolic acid linked to tyrosol (4-hydroxyphenylethanol), p-HPEA-EA: ligstroside aglycone 4-hydroxyphenylethanol-elenolic acid, 3,4-DHPEA-EA: oleuropein aglycone 3,4-dihydroxyphenylethanol elenolic acid.

*indicate that there is no agreement about the assignment of these signals
